# The Potential Health Risks and Benefits of Progesterone in the Transgender Woman Population—A Narrative Review

**DOI:** 10.3390/jcm13226795

**Published:** 2024-11-12

**Authors:** Simone Szymczyk, Katarzyna Mączka, Lidia Mądrzak, Monika Grymowicz, Roman Smolarczyk

**Affiliations:** 1 Department of Gynecological Endocrinology, Clinical Hospital of Duchess Anna Mazowiecka, Medical University of Warsaw, 2 Karowa Street, 00-315 Warsaw, Poland; katarzyna.maczka@wum.edu.pl (K.M.); lidiamad87@gmail.com (L.M.); monika.grymowicz@wum.edu.pl (M.G.); roman.smolarczyk@wum.edu.pl (R.S.); 2 Doctoral School, Medical University of Warsaw, 02-091 Warsaw, Poland

**Keywords:** transgender, progesterone, hormone, feminization, GAHT, trans, MTF

## Abstract

Introduction: Currently, progesterone is notably absent from conventional feminizing hormone therapies for transgender women. Anecdotal reports indicate the potential for health advantages following the incorporation of progesterone into treatment regimens. The primarily female hormone, progesterone naturally surges in women during the menstrual luteal phase. When administered exogenously, it may expedite bodily changes that are pivotal for gender transition. Progesterone holds promise as a potential remedy for various health conditions prevalent in the transgender woman population. Methods: This narrative review synthesizes existing literature and presents a comprehensive account of the administration of exogenous progesterone in transgender women. A literature search was conducted using the PubMed, Embase, ScienceDirect, and ResearchGate databases. The following keywords were used in the search: progesterone, transgender, breast neoplasms, lactation, prostate, testicular neoplasms, and thrombosis. These terms were combined using Boolean operators. The results of the initial search were screened by three independent reviewers based on their relevance to the topic under study. Results: A total of 104 studies were initially identified as meeting the criteria for inclusion. Following an assessment based on the contents of the title, abstract, and full text, 39 studies were deemed eligible for inclusion. A critical examination of health outcomes was conducted across key sections, including breast development, mental health, lactation, cancer risk (breast and prostate), thrombosis, and nervous and other systems. Discussion: The use of progesterone in the transgender woman population is a topic that has yet to be sufficiently researched. The limited sample size, short follow-up periods, and lack of randomization restrict the potential for achieving a robust scientific evidence base. In order to gain a fuller understanding of this topic, findings from studies on contraception, hormone replacement therapy, and animal models were considered. Conclusions: Progesterone may have a beneficial effect on the bodies of transgender women without significant adverse health effects. Further investigation through well-designed studies is recommended. Randomized controlled trials that include various dosages, broad and long-term effects, and precise demographics are needed. There is an immediate need for more knowledge to create appropriate patent and clinical practice guidelines.

## 1. Introduction

Sex and gender are fundamental aspects of human identity, each playing a critical role in how individuals experience the world. Sex is a biological characteristic determined by specific traits rooted in anatomy, physiology, genetics, and hormones. These traits typically classify individuals as male or female, although variations exist that do not fit neatly into these binary categories. In contrast, gender is a complex, multidimensional construct that encompasses a person’s identity, roles, and experiences as shaped by social and cultural expectations, behaviors, and norms [1].

While sex is frequently designated at birth based on discernible physical characteristics, gender identity is a profoundly personal phenomenon that may or may not correspond with one’s assigned sex. For a significant proportion of individuals, there is a compatibility between their sex and gender identity. Nevertheless, some individuals report a profound sense of incongruence between these aspects of their identity. This incongruence is of particular consequence in the lives of transgender individuals [2].

Among transgender populations, transgender women—who are assigned male at birth but identify and live as women—navigate a distinct set of challenges and experiences that intersect with biological, psychological, and social dimensions. These challenges are frequently exacerbated by societal judgments and expectations based on both their biological sex and their gender identity. In order to gain an accurate understanding of the experiences, health outcomes, and social dynamics affecting transgender women, it is necessary to adopt a multidisciplinary approach that takes into account the complex interplay between biological factors, identity, and societal norms [3]. An individual who identifies as transgender experiences a discrepancy between their gender identity and the gender assigned to them at birth. The term MTF, an acronym for “male-to-female”, is used to describe an individual whose innate sense of self is more aligned with femininity than the male gender assigned to them by society. Such individuals frequently pursue physical alterations that align with the characteristics typically associated with the female sex [4].

Gender-affirming hormonal therapy (GAHT) represents a viable avenue for attaining a feminine identity. The typical course of therapy entails the administration of estrogen and anti-androgens. The alleviation of gender incongruence is achieved through the inhibition of testosterone-induced effects and the promotion of estrogen-mediated alterations. These changes encompass the development of breast tissue, redistribution of body fat, diminished frequency of erections, reduced libido, slower hair loss, softer and less oily skin, testicular atrophy, decreased muscle mass, and reduced body hair. These transformations are the result of estrogenic influences and the absence of testosterone [5].

Progesterone, a vital steroid hormone, is secreted in males primarily by Leydig cells in the testes, with its production being mediated by luteinizing hormone (LH). The zona reticularis cells of the adrenal cortex are responsible for the synthesis of progesterone from cholesterol within the mitochondria, as part of the metabolic pathway of sex steroid hormones [6]. It is noteworthy that the serum concentration of progesterone in males is analogous to that observed in females during the follicular phase of the menstrual cycle. This hormone exerts a substantial impact on a multitude of physiological processes within the male body, operating through both genomic and non-genomic pathways [7,8].

The genomic actions of progesterone involve its interactions with intracellular progesterone receptors A (PRA) and B (PRB), which are widely expressed across different cell types and organs. Once progesterone binds to these receptors, it is capable of altering gene expression, thereby influencing cellular functions. In the central nervous system (CNS), progesterone functions as a neurosteroid, modulating neural activity and potentially influencing mood and behavior. Additionally, evidence suggests a potential role for progesterone in the development of certain tumors within the CNS, including meningiomas and fibromas. Progesterone’s ability to inhibit gonadotropin secretion has been demonstrated to impact spermiogenesis, underscoring its influence on male fertility. Moreover, this hormone modulates immune responses, influences respiratory function, and plays a significant role in the physiology and pathology of the prostate, including conditions such as benign prostatic hyperplasia (BPH) and prostate cancer. Furthermore, progesterone has been linked to increased appetite and weight gain, potentially due to its central effects on metabolism and food intake regulation [9,10].

Furthermore, progesterone exerts rapid, non-genomic actions. It plays a role in regulating apoptosis and the expression of progesterone receptors (mPRs), with progesterone membrane receptor components (PGRMCs), and has been linked to the estrous cycle, as well as the development of ovarian and breast cancer [11]. Progesterone plays a pivotal role in the capacitation of sperm and the acrosome reaction, which are essential processes for fertilization. Progesterone exerts an influence on the expression of luteinizing hormone (LH) receptors on the membrane of Leydig cells, which in turn affects the biosynthesis of testosterone. In the prostate, progesterone has the potential to increase the concentration of its receptors, which may subsequently influence prostate function and pathology. Moreover, progesterone exerts effects on kidney function by modulating fluid balance and impacts adipose tissue by influencing fat storage and metabolism. Additionally, as a neurosteroid, it plays a role in modulating neural activity through non-genomic pathways [9,12].

The objective of this study was to examine the physiological alterations induced by exogenous progesterone in male-to-female (MTF) transgender patients, with a particular emphasis on its impacts on breast development, male reproductive organs, the central nervous system, and hemostasis. The aim was to gain a comprehensive understanding of how progesterone influences these structures and systems in MTF patients undergoing hormone therapy. Progesterone may possess an enigmatic nature that, despite being anecdotal, could demonstrate satisfactory effects when added to the transition treatment plan within the MTF community. The potential for progesterone to serve as an additional, enhancing agent to the primary role of estrogen remains a subject of ongoing investigation.

This review places particular emphasis on the role of oral micronized progesterone (MP) as a progestogen used in the transgender woman population. The chemical structure of oral micronized progesterone (MP) is identical to that of the progesterone produced by the human ovary. It has been demonstrated to be an effective method of endometrial protection in hormone replacement therapy (HRT) during menopause. In contrast to medroxyprogesterone acetate, which has been associated with an elevated risk of cardiovascular events and breast cancer, MP is metabolically neutral. This indicates that it does not negate the beneficial effects of estrogen on lipid metabolism and does not appear to elevate the risk of breast cancer or cardiovascular disease, although further data are required to substantiate this [13,14,15,16,17]. Furthermore, oral MP has been shown to confer benefits for patients with endometrial hyperplasia (EH). Nevertheless, it is not generally considered the preferred option for progestin therapy in women [18]. While our principal objective is to examine the influence of oral MP, we also incorporated data from individuals who are taking other progestogens, including medroxyprogesterone and cyproterone acetate. These latter agents are frequently utilized as a primary anti-androgen for MTF patients. Given the ongoing evolution of Gender-affirming hormonal therapy (GAHT) for transgender individuals, there is a pressing need for further research in this area. The existing reviews on the use of progesterone in male-to-female (MTF) transgender patients frequently fail to encompass the comprehensive range of effects and considerations associated with this hormone. This study employs a comprehensive, multidisciplinary approach to address this topic. A comprehensive review that synthesizes findings from various study designs and provides a critical assessment of the existing literature would offer a more nuanced perspective. Such an approach could identify precise research directions, thereby establishing a foundation for more rigorous, targeted studies on progesterone use in MTF hormone therapy. It is imperative that this literature gap be addressed in order to advance knowledge, refine clinical guidance, and ultimately enhance the quality of care for transgender individuals seeking gender-affirming treatment.

**Figure 1:**
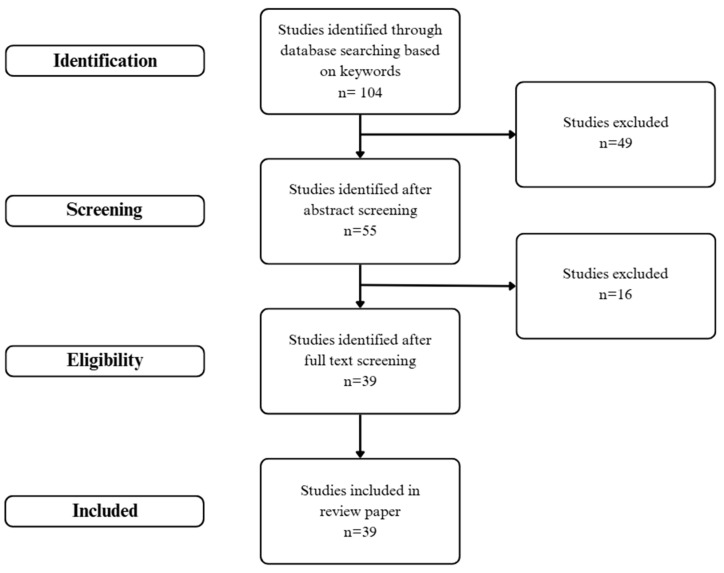
Flow diagram of study selection.

A comprehensive literature search was conducted from May to November 2024 using the PubMed, Embase, ScienceDirect, and ResearchGate databases. The review process entailed several pivotal stages, including the selection of studies and the categorization of themes. The objective of the search strategy was to identify studies that explored the use of progesterone, specifically oral MP, in transgender women. The search terms included a combination of Medical Subject Headings (MESH) and free-text terms, such as “progesterone”, “transgender”, “breast neoplasms”, “lactation”, “prostate”, “testicular neoplasms”, and “thrombosis”, used both independently and in conjunction with one another. To guarantee the relevance and quality of the studies included in this review, three independent reviewers evaluated the results of the preliminary search. The selection process entailed a review of the titles, abstracts, and full texts of the studies to ascertain their relevance and contribution to the review’s objectives. In order to be included in the review, studies had to focus on the usage of MP in transgender women and provide data on the effects of this medication, potential complications, and underlying physiological mechanisms. The analysis excluded book chapters, duplications, editorial letters, and proceedings. The reference lists of selected meta-analyses, systematic reviews, clinical studies, and case studies were manually screened for further identification of relevant studies. The flow diagram of the study selection process is illustrated in Figure 1, and the reviewed studies are presented in Table S1.

Table S1 in the Supplementary Materials provides a summary of the reviewed literature.

## 2. Breast Growth

One of the primary motivations for initiating hormone therapy among MTF patients is the potential for breast development. Nevertheless, a considerable number of transgender women express discontent with the conventional estrogen and anti-androgen therapies, frequently contemplating surgical breast augmentation as an alternative [19]. This inclination toward surgical intervention may potentially give rise to a number of complications, which could be mitigated by progesterone-supported hormonal treatments. Gelles-Soto D. et al. discuss the use of progesterone in conjunction with feminizing chest surgery [20]. Anecdotal evidence suggests that progesterone may contribute to maturation of the breast and an increase in tissue volume [21]. Nevertheless, research indicates that administering oral MP prematurely may impede potential growth [22]. A literature gap prompted investigations into the potential benefits and risks of exogenous progesterone supplementation with oral micronized progesterone (MP) in male-to-female (MTF) patients with regard to breast development.

In the study conducted by Bahr et al., a total of 29 participants were randomly assigned to receive one of three forms of progesterone: twenty-five were administered oral micronized progesterone (MP), two received MP rectally, and the remaining two were given intramuscular medroxyprogesterone. Transgender women who underwent progesterone-enhanced feminizing hormone therapy reported increased satisfaction with their breast development at six and nine months of treatment. The proportion of patients who were satisfied with their breast development was significantly higher in the progesterone-enhanced hormone therapy group than in the control group at both six and nine months (53.8% vs. 19.6%; *p* = 0.004) (71.4% vs. 20.8%; *p* = 0.003) [23]. Another study involved 20 MTF patients and sought to ascertain the impact of exogenous progesterone supplementation with oral MP. However, over the course of the three-month study period, no statistically significant changes were observed between the self-reported Tanner stage evaluations for the two groups (progesterone vs control group 3.5 (3.2, 3.7) vs 3.6 (3.3, 3.9) *p* = 0.42) [24]. It is important to note that the sample size of the study was relatively small (n = 20) and that the duration may have been insufficient, given that standard breast development can continue for up to three years [25]. It is not possible to make a reliable comparison between these two studies. The two studies employ disparate timeframes and scales for measuring disparate outcomes. A randomized controlled trial is currently underway to investigate the potential for breast growth in the male-to-female (MTF) population under the influence of estradiol and oral micronized progesterone (MP) therapy, including the administration of various hormone doses. It is noteworthy that this research employs reliable measures of breast volume, which is a notable omission in previous studies in this field. Three-dimensional scans offer a more objective means of measurement than satisfaction and self-reported Tanner scales questionnaires [26]. Dijkman B. A. M. et al. present a case of a patient with complete androgen insensitivity syndrome who experienced fluctuations in breast size based on the prescribed hormone therapy. Following a period of estrogen monotherapy, the introduction of progesterone resulted in a 17% increase in breast volume [27]. While the impact of estrogen is well-documented, the role of progesterone in this context requires further investigation and scrutiny due to the comparatively lesser extent of research in this area. A recent study examining the effects of gender-affirming hormonal therapy (GAHT) on breast growth volume and satisfaction excluded women who used progesterone. It will not be included in the subsequent analysis [28].

## 3. Mental State

The extant evidence suggests that progesterone may exert an influence on human cognition, with specific effects on memory and cognitive processing. This is observed by Henderson et al. [29], who highlights its role in modulating cognitive functions in various populations. Furthermore, self-estimated intelligence, perceptions of mental state, working memory, creativity, and psychological correlates are influenced by a complex interplay of factors, as examined in studies by Giannouli et al. [30] and Reilly et al. [31]. These studies demonstrate that sex differences frequently manifest in self-assessed cognitive abilities, with males exhibiting a tendency towards overestimation and females towards underestimation. This phenomenon is sometimes described as “male hubris, female humility”. For transgender individuals undergoing hormone therapy, including feminizing regimens that include progesterone, there is the potential for hormone-related shifts in cognition and self-perception. However, the nature and extent of these effects remain underexplored. Further research into the impact of progesterone on self-assessed intelligence and mental state in transgender populations could enhance our comprehension of the cognitive and psychological aspects of gender-affirming hormonal therapy (GAHT).

It is hypothesized that the marked decline in progesterone serum levels that occurs following childbirth is a key pathophysiological factor in the etiology of postpartum depression and psychosis. S. Trifu et al. [32] propose that hormonal balance may be a significant factor influencing the mental health of transgender individuals. A synthesis of endocrinological and psychiatric perspectives on mental health may offer a novel avenue for future research. Further research is required in the form of randomized controlled trials with measured exposure to fluctuating progesterone levels, with a particular focus on cognition and self-estimation of mental status.

With regard to mental health outcomes, there is a paucity of published research that specifically addresses the influence of progesterone on libido within the transgender population. A substantial proportion of the transgender population may experience sexual dysfunction, including diminished libido, difficulties in achieving orgasm, and distressing apprehensions regarding sexual contact. A total of 69% of MTF individuals reported at least one sexual dysfunction (n = 246) [33]. These challenges may have their roots in gender incongruence, psychological factors, and potentially in hormonal imbalances. Progesterone has emerged as a potential avenue for addressing these complex concerns.

The transgender community has articulated the beneficial effects of progesterone; nevertheless, randomized placebo-controlled trials remain absent in this domain. The results of studies conducted on cisgender women indicate that increased progesterone levels can diminish libido [34]. Nevertheless, extrapolating these findings to the MTF population remains uncertain and thus warrants further investigation.

Another area of interest regarding progesterone administration is its potential to enhance sleep quality and stabilize mood. It is generally observed that the MTF population presents with a lower quality of sleep and an increased prevalence of mental health issues when compared to the general population [35]. The combination of hormone treatment with additional support, such as progesterone, has been theorized to offer therapeutic benefits. However, a study by Nolan, B.J., et al. addressing this matter found no statistically significant differences (*p* = 0.35) using the PSQI scale [24]. The limitations of this study included the use of a small sample size (n = 19 vs. n = 17), a relatively short follow-up period (three months), and an absence of randomization. It is noteworthy that higher doses of progesterone (100 mg) demonstrated a slight improvement, which has prompted speculation about potential effects on sleep. However, this difference was not statistically significant when compared to the control group (n = 4, *p* = 0.84). Furthermore, there is no evidence to suggest that it has an impact on mental health. Despite the observed reduction in psychological distress after three months, the progesterone group exhibited a slight improvement, measured on a K10 scale with an average score of 22.5 compared to 24.4, while the control group showed a similar trend, with an average score of 23.8 compared to 25.2. However, these changes were not statistically significant (*p* = 64) [24]. The aforementioned study by Bahr, C. et al. examined patient changes in mental state. It was observed that mental illness severity at six months improved in patients receiving progesterone alongside standard gender-affirming hormonal therapy (GAHT) *p* = 0.009. However, this effect did not persist until nine months (*p* = 0.19). No further differences were identified. No statistically significant differences were observed in libido, testosterone suppression, or weight changes [23]. Morssinkhof M. W. L. et al. demonstrated that 12 months of gender-affirming hormonal therapy (GAHT) had no impact on self-reported sleep quality in 183 transgender women. Progesterone is referenced in the study, yet no statistical interpretation of its effect on the population is provided [36].

It is advisable to exercise caution when selecting an appropriate dosage. Although the mood-stabilizing effects of progesterone have yet to be substantiated, it is noteworthy that two patients in this study withdrew due to the exacerbation of symptoms. Kalayjian, A. et al. conducted a retrospective cross-sectional study to investigate the potential for clinically significant drug–hormone interactions between progesterone and psychotropic medications. No data regarding progesterone is presented. Transgender patients are stated to be at high risk of psychiatric polypharmacy, with 68 patients (37.0%) out of 184 participants exhibiting this phenomenon [37]. Additionally, a potential side effect of progesterone includes the induction of depressive symptoms, as observed in cisgender woman populations. The available evidence from studies conducted in cisgender women indicates that progesterone may have an impact on sleep quality and mental health. This highlights the need for further research and careful consideration of the potential effects of progesterone in the transgender population. The currently available data do not support the implementation of progesterone in the hormonal regimen of MTF patients for the purpose of improving mental state, libido, and sleep quality.

## 4. Lactation

Lactation is defined as the production of milk by mammary glands. It is a process that has been identified as a potential unintended outcome of gender-affirming hormonal therapy (GAHT) [38]. Nevertheless, there is a specific patient cohort for whom this bodily function is desired. Some individuals who are transitioning from one gender to another seek to breastfeed their children, with the aspiration of fulfilling maternal roles and forging deeper bonds with their offspring. The efficacy of a tailored progesterone regimen in facilitating this process as part of hormonal therapy was demonstrated in several case studies.

In two case studies, patients were administered an incremental increase in oral MP dosage, from 100 mg to 200 mg, in conjunction with the galactagogue domperidone at a dose of 10 mg three times a day, with dosage adjustments made to achieve optimal efficacy. In another instance, the dosage of progesterone was increased to 400 mg per day. The milk produced by these methods was found to be comparable to that of cisgender women, providing sufficient quantities to nourish a newborn [39,40]. It is noteworthy that breast milk from MTF mothers may offer superior benefits, including an enhanced immunological profile, compared to artificial formula [41]. The composition of breast milk from MTF patients has been presented by Ikebukuro et al. in a recent study. The composition of the milk produced by MTF mothers has been found to be comparable to that of milk produced by women at 7–8 months of gestation, as determined by analysis conducted at the 199th–215th day of treatment. Additionally, the milk has been observed to contain a high concentration of secretory immunoglobulin A, which suggests that it may possess immunological functions. [42]. Delgado et al. developed a proposed lactation induction protocol and analyzed human milk parameters; however, the full text was not available for review [43].

A single case report [44] demonstrated the successful induction of lactation through the administration of 0.4 mg/72 h transdermal estradiol, 300 mg of MP daily, and 10 mg of metoclopramide three times daily. It is noteworthy that previous case reports do not include the use of domperidone. In the fourth case, although the patient produced milk, supplementary feeding was required. Furthermore, the authors presented recommendations and a proposed treatment regimen for the induction of lactation [45].

A deeper comprehension of the role of progesterone in lactation is of paramount importance for future MTF mothers. Progesterone induces the proliferation of mammary glands and an increase in ductal tissue volume, thereby preparing the mammary glands for lactation. The introduction of prolactin, a key hormone necessary for lactogenesis, promotes the maturation of milk production and prioritizes β-casein secretion pathways in mammary epithelial cells [35,46]. Subsequently, the withdrawal of progesterone serves as a trigger for the onset of a secretion mechanism that mimics the hormonal changes that occur following childbirth. Furthermore, elevated progesterone levels in the bloodstream do not impede the onset of lactogenesis [47,48].

The administration of exogenous MP has been identified as a promising solution to enhance mammary gland development and function in MTF patients. The provision of healthcare services that encompass a range of treatment modalities, selected on the basis of the specific requirements of each patient, has the potential to markedly enhance satisfaction with treatment and to mitigate the distress associated with gender dysphoria among transgender individuals.

## 5. Prostate Cancer

In a case report by Sharif A. et al. [49], prostate cancer was linked to exogenous estrogen treatment, raising concerns within the transgender community. It has been theorized that progesterone may act as a counterbalancing agent capable of reversing these adverse effects. A study conducted by Ingham and colleagues demonstrated the presence of estrogen and progesterone receptors on prostate cancer cells, which exhibited responsiveness to pharmacological stimulation. While estrogen has been demonstrated to promote cell division, progesterone has been shown to exert an inhibitory effect on this process [50]. Nevertheless, research explicitly delineating the potential risks of prostate cancer in transgender women using progesterone remains scarce.

There is a paucity of knowledge regarding the prostate and its associated health risks within the transgender community. The primary effect of gender-affirming surgeries (SRSs) is the repositioning of the prostate due to the creation of a neovagina anteriorly, which alters the standard palpation site. The advent of novel anatomical structures consequent to surgical procedures presents a challenge to the conventional surgical and radiotherapeutic techniques employed in the management of prostate-related issues. Moreover, prolonged hormonal therapy frequently results in prostate atrophy, which leads to a reduction in prostate size and, consequently, makes examinations more challenging [51].

Furthermore, a significant number of urologists lack the requisite experience in addressing the specific medical concerns of transgender women. This has resulted in the implementation of inadequate screening protocols and health campaigns that tend to overlook this population. The distinctive anatomical alterations and healthcare requirements of transgender women necessitate tailored attention and adapted clinical practices to guarantee comprehensive and suitable medical care. It is imperative that these distinctive challenges be given greater attention and that specialized care protocols be established.

## 6. Thrombosis

As with any pharmaceutical agent, progesterone has the potential to induce adverse effects. Among the adverse effects commonly observed in the MTF population are headaches, while less frequent effects include fluid retention, somnolence, dizziness, gastrointestinal issues, rash, acne, and breast tenderness. Nevertheless, the most significant concern that merits emphasis is the potential risk of thrombosis [52].

Thrombosis represents one of the most prevalent risks associated with gender-affirming hormonal therapy (GAHT). Estrogen, a primary component in hormone therapy, has been extensively identified as a significant risk factor for complications in medical treatments. Despite the lack of the explicit inclusion of contraceptives in risk scales such as the Wells Scale, evidence suggests that hormonal birth control can pose a significant risk, with estimates indicating a 6% increased risk over a 10-year period [53]. It is not straightforward to extrapolate the knowledge concerning hormonal contraceptives and thrombosis risk to the case of hormonal therapy with 17β-estradiol and micronized progesterone. However, it should be noted that MTF patients often use hormonal treatments for extended periods of time, which may elevate their overall risk compared to cisgender women [54].

For patients with an elevated risk profile, transdermal estrogen administration, which is regarded as a relatively low-risk approach, is typically advised [55]. However, there is a notable absence of research specifically focusing on the risk of deep vein thrombosis (DVT) when adding progesterone to standard MTF gender-affirming hormonal therapy (GAHT). Based on studies examining the combined use of estrogen and progesterone in menopausal hormone therapy for cisgender women, it is reasonable to conclude that the addition of oral micronized progesterone (MP) does not significantly elevate the risk of thrombotic complications [48,56]. It is important to note, however, that oral MP exerts a comparatively lesser impact on (DVT), homeostasis, and blood pressure compared to gestagens utilized in menopausal hormone replacement therapy. The effects of gestagens on body weight, cholesterol levels, and carbohydrate metabolism were not observed. Furthermore, in contrast to gestagens, no significant increase in the relative risk of developing breast cancer was observed [57].

A further case study illustrated that the cessation of progesterone and the modification of estrogen administration resulted in a reduction in the risk of deep vein thrombosis (DVT) in a patient who had previously been subjected to excessive progesterone administration. In their study, Boskey and colleagues conclude that a careful balance must be maintained between assessing the risk of thrombotic complications and the potential emotional consequences of discontinuing hormonal therapy. In light of the inconsistency and limited scope of the available data, it is advisable to adopt a cautious approach, including the evaluation of (DVT) risk and its juxtaposition with other relevant factors. In the absence of further comprehensive investigation, the optimal course of action remains undetermined [58].

Although the existing literature is limited, a growing body of evidence suggests that patients undergoing external progesterone therapy may be at an elevated risk of postoperative thrombosis. This underscores the importance of vigilant patient monitoring, comprehensive risk disclosure, and recommendations for preventive measures when initiating progesterone treatment. Prophylactic measures, as proposed by de Barros et al. [59], include the administration of anticoagulant medication and the cessation of hormonal therapy for a period of two to four weeks prior to the performance of immobilizing surgery. It is inadvisable to recommend progesterone as a supportive hormone to GAHT for patients at elevated risk of DVT. Future prospective randomized controlled trials focusing on the thrombogenic effects of progesterone could address this pressing literature gap. It is recommended that close monitoring of progesterone and estrogen serum concentration and administration route, as well as thorough reporting of the incidence of thrombotic complications, be included in any future studies. It would be beneficial to consider the population of MTF individuals undergoing sex reassignment surgeries as a subject for further investigation.

## 7. Breast Cancer

One critical aspect regarding the impact of progesterone on the human body is its possible association with an increased risk of developing breast cancer. A retrospective nationwide cohort study by de Blok et al. demonstrated a notable increase in the incidence of breast cancer among transgender women undergoing hormonal therapy. Studies focusing on breast cancer among MTF individuals indicate an elevated risk compared to cisgender males (standardized incidence ratio 46.7, 95% confidence interval 27.2 to 75.4) and a slightly lower risk compared to cisgender females (0.3, 0.2 to 0.4). A total of 17 patients out of 2260 MTF individuals were identified. Progesterone receptors were identified in eight of the twelve tumors. It is unclear from the available data whether the patients were taking oral MP [60].

The analysis of gender differentiation syndromes such as Klinefelter syndrome, in which the Y chromosome exerts protective effects against breast cancer, underscores the potential for discernible distinctions in patient populations [61]. An additional distinguishing factor is the absence of hormonal monthly cycles in transgender women, which may contribute to an altered breast cancer risk profile. Research indicates that fluctuations in progesterone levels are a significant contributing factor in the progression of breast cancer [62]. The characteristics of breast cancer in transgender women consistently demonstrate the presence of progesterone-positive cancers [63].

In considering the prescription of progesterone, it is of the utmost importance to ascertain the patient’s family history of breast cancer, specifically whether the cancer in question was progesterone-positive. It is imperative that healthcare providers remain vigilant about the available breast cancer prevention methods that are tailored to transgender women. In light of the fibrous characteristics of transgender women’s breasts, breast ultrasound may be a more optimal diagnostic option. Furthermore, breast augmentation in some patients may potentially compromise the efficacy of mammography methods [64].

## 8. Brain

In the scientific community, there is a drive to obtain definitive evidence concerning the structural brain changes that underpin gender incongruence. A review of the literature on the impact of altered hormone profiles on neuroanatomy revealed some intriguing findings. The administration of exogenous estrogen was observed to result in a reduction in grey matter volume and hippocampal volume in the brain, which had previously been dominated by testosterone. Furthermore, these structural alterations were accompanied by an increase in ventricular volume. However, the addition of cyproterone acetate as a progestogen to newly prescribed estrogen hormonal therapy resulted in a reduction in the magnitude of these changes. It is noteworthy that the most significant changes often manifest during the neonatal stage [65,66]. Handschuh PA. et al. demonstrated that gender-affirming hormonal therapy (GAHT) in transgender individuals resulted in alterations in grey matter density and microstructure of various brain regions. One of the hormones studied was progesterone, and no direct link has yet been established between its effects and the results observed [67].

Progesterone is postulated to exert neuroprotective effects, as evidenced in animal models where it is implicated in memory and cognitive functions [68]. The use of gender-affirming hormonal therapy (GAHT) has been linked to an exacerbation of symptoms associated with migraines and epilepsy. Nevertheless, while natural progesterone has demonstrated neuroprotective effects that could potentially reduce the frequency of attacks, the synthetic medroxyprogesterone, which is commonly used in gender-affirming treatments, does not offer the same benefits.

Although there is currently no definitive evidence regarding the transgender population, studies performed on cisgender women suggest that the combination of progesterone and estrogen administration may influence cerebral vessels, potentially increasing the incidence of attacks and their negative consequences [69].

Martinez et al. propose that both progesterone and estrogen play a role in the pathophysiology of migraines. The influence of progesterone is particularly noteworthy, as it has been demonstrated to both induce and inhibit migraine attacks, depending on its concentration levels [70]. The authors put forth the proposition that progesterone may serve as a prospective prophylactic agent for migraines, contingent on the maintenance of stable levels. The neuroprotective effects of progesterone are primarily directed towards the prevention of cortical hyperexcitability and the modulation of inhibitory signaling via gamma-aminobutyric acid (GABA) neurons. Progesterone has been demonstrated to result in a reduction in brain-derived neurotrophic factor (BDNF) and other neurotrophins, which are associated with neuronal growth and hyperexcitability when exposure occurs over an extended period. Progesterone has been demonstrated to reduce serotonin synthesis and decrease monoamine oxidase (MAO) expression. These effects may impact migraine mechanisms, as serotonin levels have been identified as a crucial factor in migraine pathology. Furthermore, progesterone has been observed to elevate the levels of pro-inflammatory neuropeptides, including calcitonin gene-related peptide (CGRP), and to stimulate the release of nitric oxide (NO), which can result in vasoconstriction and the onset of an attack [70,71].

Further research is required to gain a deeper understanding of the combined effects of progesterone and estrogen administration in MTF patients. The transgender woman population offers a distinctive opportunity to investigate the potential pathophysiological role of progesterone in various neurological disorders.

## 9. Other Organs

Progesterone exhibits a wide range of receptor expressions across various tissues, which appear to be unrelated to conventional gender-related functions.

It is noteworthy that progestogens have been observed to possess potential protective properties against the degenerative impact of excess estrogen on ocular structures [72].

A case study, as described by Jackson Cullison et al., presents a patient with porphyria who experienced an exacerbation of symptoms subsequent to the administration of excessive quantities of estradiol and progesterone. The cessation of therapy resulted in the reversal of these effects, thereby suggesting a potential role for MP in influencing negative outcomes [73].

A number of studies have indicated that the concurrent administration of estrogen and progestogens may potentially attenuate the progression of symptoms associated with SARS-CoV-2 infection [74]. The impact of progesterone on the prevalence of DVT may influence the potential complications associated with the infection. In vitro studies have demonstrated that progesterone reduces the expression of ACE2 receptors in testicular tissue. The precise impact of progesterone on the transmission of the SARS-CoV-2 virus remains unclear [75].

Furthermore, progesterone has been linked to augmented fibrotic effects on testicular structures and Leydig cell atrophy, which may result in substantial and potentially irreversible impacts on fertility [75]. It is of the utmost importance to consider fertility-preserving techniques for interested patients prior to the administration of progestogens.

The pancreas is another organ that is influenced by progesterone, which has the potential to enhance glucose tolerance. The results of studies conducted on mice indicate that beta cells in the pancreas respond to progesterone, resulting in alterations to metabolic processes. However, these effects may be influenced by the presence of other hormones, and conclusive human in vivo implications remain uncertain [76,77]. A recent case study presented a patient who was taking oral MP and subsequently developed gallstone-associated acute pancreatitis. The authors hypothesize that estrogen may contribute to stone formation [78]. Further research could investigate whether patients taking progesterone may be at an increased risk of experiencing adverse effects from their medication.

In consideration of the liver’s role in detoxification and drug metabolism, prolonged exposure to progesterone may potentially impact its function. In the liver, progesterone is converted into estradiol and subsequently combined with glucuronic acid prior to excretion in the urine. Despite the absence of dedicated research on the MTF population, existing evidence indicates that progesterone may serve to mitigate the impact of developing a fatty liver. However, it has been linked to unfavorable outcomes in conditions such as hepatocellular carcinoma (HCC), bile duct diseases, and the progression of hepatitis E virus (HEV) infection. Progesterone may play a role in causing metabolic injury in the liver [79,80].

Furthermore, it is possible that progesterone may influence the outcomes of gender-affirming surgical procedures. Hormonal therapy has the potential to affect peripheral nerves, which may increase the risk of injury, inhibit regeneration, and cause vulnerability to ischemic changes. The potential pathophysiology of postoperative neuropathy in transgender individuals is presented for theoretical discussion [81].

The latest research on MTF healthcare takes into account the prevalence of progesterone usage within the community. Stelmar J. et al. provide a comprehensive description of the alterations in serum progesterone levels observed in patients who underwent a gender-affirming bilateral orchiectomy [82]. In a cohort study conducted by Moghadam et al., computed tomography scans were performed on 45 patients to evaluate the influence of progesterone in the redistribution of facial fat volumes. The findings indicated that progesterone does not exert a substantial influence on this process [83]. The work of Lachlan M. Angus and colleagues demonstrates that there are still unique avenues to be explored in transgender research [84]. The subsequent phase of this research may encompass the administration of oral MP in measured doses, with subsequent observation of QTc interval alterations.

**Figure 2:**
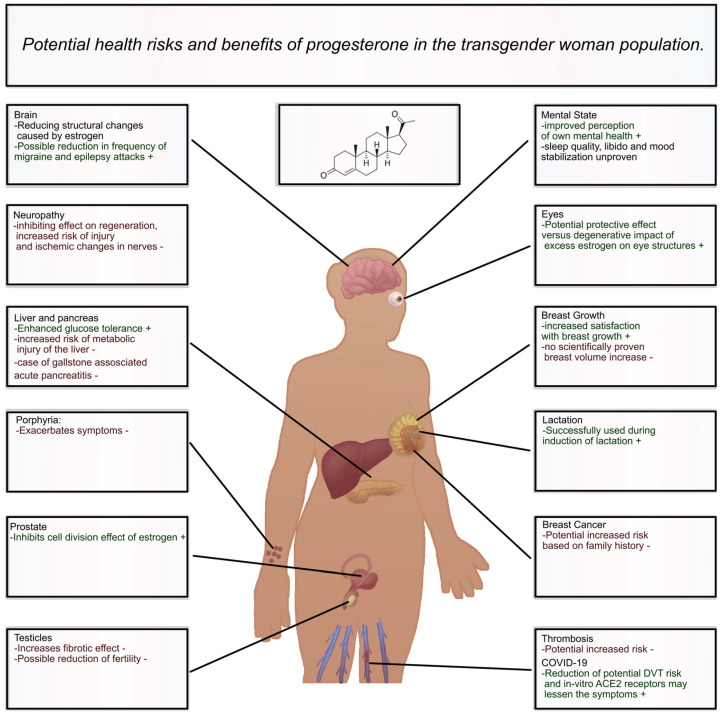
The effects of progesterone on diverse body systems in MTF patients.

A summary of the effects of progesterone on the physiology of MTF patients is presented in Figure 2.

## 10. Conclusions

The exploration of progesterone treatment in the MTF population is still significantly under-researched. The existing studies often have limitations, including inadequate population sizes, short follow-up periods, and a lack of blinding. The number of prospective studies and meta-analyses on this topic remains insufficient. A significant proportion of the presented research is based on assumptions derived from the extrapolation of findings from studies on female contraception or from animal and in vitro models. It is not possible to determine the long-term effects and potential chronic health impacts of progesterone in the MTF population from the current compilation of studies. The potential health risks and benefits presented here are based on a limited number of studies, often with preliminary findings. To ensure the reliability of any conclusions drawn from the current body of research, there is a pressing need for comprehensive long-term prospective studies that are specifically focused on the transgender community.

It is crucial to acknowledge that progesterone should serve a supplementary function in gender-affirming hormonal therapy (GAHT) for individuals who identify as male-to-female (MTF). The primary agents responsible for the desired physical changes during the transition process are estrogen and anti-androgen medications. Therefore, progesterone should not be used as a replacement for these core treatments. Nevertheless, progesterone’s supportive role has the potential to enhance patient satisfaction and may contribute to more personalized approaches that address the diverse needs of transgender individuals seeking gender-affirming care. The extensive effects of progesterone on the entire organism may extend beyond gender-related concerns, potentially serving as a preventive or adjunctive measure in the treatment of ailments unrelated to gender identity.

It is important to note that this review does not provide a definitive basis for advocating the incorporation of progesterone into the gender-affirming hormonal therapy (GAHT) regimen for male-to-female (MTF) patients. The current body of evidence does not provide sufficient support for such a recommendation. Nevertheless, it is noteworthy that no substantial risks that would outright preclude further investigation have been identified. Patients frequently ingest oral doses of progesterone ranging from 100 to 200 mg. The occurrence of serious adverse effects is not frequently documented in existing literature. Nevertheless, individuals who self-medicate are advised to maintain comprehensive health records. In the event of the emergence of any novel adverse effects, it is imperative to discontinue the medication and promptly seek medical counsel. The studies presented herein establish a foundation for more comprehensive and robust scientific investigations that could yield insightful outcomes. Further expansive and meticulously designed scientific experiments are required to elucidate the potential role and clinical implications of progesterone in MTF hormone therapy, thereby paving the way for informed and evidence-based healthcare decisions in transgender health. A holistic approach to transgender medicine is recommended, with a focus on the overall well-being of the patient. It is recommended that new studies be designed as large, prospective, and randomized controlled trials. The participants should be divided into groups according to their oral progesterone dosage: a control group with no progesterone and two groups taking either 100 mg or 200 mg. A further division should be made between those who have completed puberty and those who commenced their transition during this period. It is recommended that breast size be measured on a regular basis using a 3D scanner. The assessment of patient satisfaction with their transition, breast growth, mood, sleep quality, weight and libido should be conducted using appropriate scales. The psychiatric health of patients can be monitored through the analysis of their medical records. It is imperative that any medical events be duly documented in order to explore potential correlations with the medication and to create new avenues for research.

## Data Availability

The data presented in this study is available on request from the corresponding author. The data is not publicly available due to privacy.

## Supplementary Materials

The following supporting information can be downloaded at: https://www.mdpi.com/article/10.3390/jcm13226795/s1, Table S1: summary of the reviewed literature.
